# Silica–Cyclodextrin Hybrid Materials: Two Possible Synthesis Processes

**DOI:** 10.3390/ijms25021108

**Published:** 2024-01-16

**Authors:** Marta Gallo, Barbara Onida, Luigi Manna, Mauro Banchero

**Affiliations:** Department of Applied Science and Technology, Politecnico di Torino, Corso Duca Degli Abruzzi 24, 10129 Turin, Italy; marta.gallo@polito.it (M.G.); barbara.onida@polito.it (B.O.); luigi.manna@polito.it (L.M.)

**Keywords:** hybrid, silica, cyclodextrin, aerogel, pollutant adsorption, drug release

## Abstract

Both cyclodextrin (CD) and porous silica possess interesting properties of adsorption and release. A silica–CD hybrid, therefore, could synergically merge the properties of the two components, giving rise to a material with appealing properties for both environmental and pharmaceutical applications. With this aim, in the present study, a first hybrid is obtained through one-pot sol–gel synthesis starting from CD and tetramethyl orthosilicate (TMOS) as a silica precursor. In particular, methyl-β-cyclodextrin (bMCD) is selected for this purpose. The obtained bMCD–silica hybrid is a dense material containing a considerable amount of bMCD (45 wt.%) in amorphous form and therefore represents a promising support. However, since a high specific surface area is desirable to increase the release/adsorption properties, an attempt is made to produce the hybrid material in the form of an aerogel. Both the synthesis of the gel and its drying in supercritical CO_2_ are optimized in order to reach this goal. All the obtained samples are characterized in terms of their physico-chemical properties (infra-red spectroscopy, thermogravimetry) and structure (X-ray diffraction, electron microscopy) in order to investigate their composition and the interaction between the organic component (bMCD) and the inorganic one (silica).

## 1. Introduction

Cyclodextrins (CDs) are a category of cyclic oligosaccharides with fascinating properties. Thanks to the chemistry of their toroidal conformation, these molecules possess a lipophilic cavity and a hydrophilic external surface, which confer a particularly versatile character to these compounds [1]. Indeed, cyclodextrins are widely used as excipients for both adsorption and release applications, since CDs can form complexes with hydrophobic molecules, thus increasing their solubility and stability. In the literature, studies related to the use of CDs, alone or in combination with other materials, cover a broad spectrum of applications. For example, CDs can be used as pharmaceutical excipients; depending on the size of the drug molecule, one or two CD molecules can complex the drug, enhancing its bioavailability. In some cases, instead, CDs can release the complexed molecule upon specific triggering signals (e.g., in the study by Silva et al. [2] an anticancer drug is released from CD–gold nanoparticles upon photo excitation). Furthermore, CD polymers, which are based on cross-linked CDs, and electrospun CD fibers are also studied in the biomedical field as drug and gene carriers [3,4]. Being recognized as safe by the Food and Drug Administration [5], CDs can be employed in the food industry to encapsulate active compounds or control the taste of food [5]. In this context, when incorporated into fibers or films, they can be used in food packaging applications such as the retention of aroma [3]. Moreover, CDs can be applied in heterogeneous catalysis, where they are coupled with metal nanoparticles [6], or as adsorbents for the removal of polluting organic molecules. As far as this last application is concerned, for example, nanosponges made of crosslinked CDs have been proven to be able to completely remove a dye (phenolphthalein) from a water-based solution [7]. Also, when immobilized on magnetic materials or fibers, they can serve as adsorbents for the removal of pollutants from wastewater [8]. When coupled with polymers instead, CDs can be used in oil fields to enhance oil recovery [9]. Finally, CDs immobilized on silica can act as chiral selectors in chromatography [10] as well as adsorbents for water remediation [11].

Another category of highly efficient carriers or adsorbents is that of porous silica. Thanks to their controllable structures and compositions, as well as the possibility to tune both their pore size and surface composition, porous silicas are suitable for various applications ranging from catalysis to chromatography, separation and adsorption. Indeed, porous silica, both pristine and functionalized, is reported to be an excellent adsorbent for molecules both in liquid and gas phases [12,13,14,15,16]. For example, silica aerogels have been proven to be efficient in removing methylene blue dye from aqueous solutions [17,18]. At the same time, porous silica is also widely studied as a delivery platform for drugs and active principles [19,20,21,22,23,24,25,26]. Moreover, in the case of topical drug release, silica can offer added value since it may release small amounts of silicic acid, which has a beneficial effect for the skin [27].

Both CDs and silica possess appealing and apparently analogous properties of adsorption and release; however, these materials have complementary functions. Unless thermally treated, silica usually has a mainly hydrophilic surface thanks to the presence of –OH groups and, hence, it is particularly efficient in adsorbing polar molecules [28]. On the other hand, the internal cavity of CDs is lipophilic and can host non-polar molecules. In addition, in view of possible applications for adsorption from solutions, it has to be remembered that generally, CDs are water-soluble molecules, whilst silica is more stable in aqueous media [29]. A silica–CD hybrid material, therefore, could offer synergic action of the two constituents, representing an interesting support to either adsorb or release hydrophilic and hydrophobic molecules simultaneously. Such a material may find interesting uses as both a pollutant remover and a drug delivery device. In addition, silica could stabilize the CD, making it an adsorbent material suitable for applications in water-based solutions, too.

From this perspective, the present work is a feasibility study that aims at investigating whether such a hybrid material made of silica and cyclodextrin can actually be obtained. At present, the literature does not report many examples of the synthesis of silica–CD hybrids [30,31,32,33]. Even though the few proposed examples of porous silica–CD hybrids are effective, their synthesis processes require the use of toxic and aggressive solvents (such as dimethylformamide and toluene [30,34]) and are time- and energy-consuming (i.e., they involve several steps at high temperatures [30]). Moreover, when a templating agent is included in the synthesis, an additional step involving the use of organic solvents and heating is required to remove the surfactant [34,35], thus resulting in an even longer and more complex process. On the other hand, hybrids obtained by simply grafting CDs on silica or by coating silica with CDs often present an uneven distribution of the CDs [30]. To avoid this problem, hybrid systems should be prepared through a sol–gel process. However, a simple and mild sol–gel process suitable for obtaining a CD–silica hybrid without using polluting solvents and high temperatures is still missing.

In this work, two one-pot and environmentally friendly synthesis routes are proposed and compared. First, a dense silica–CD hybrid material is prepared through a sol–gel process in an acid aqueous solution where a CD (methyl-β-cyclodextrin, bMCD) is used together with TMOS. Afterwards, a second synthesis process is optimized in order to obtain silica–bMCD hybrids in the form of aerogels. Aerogels are a class of materials that have elevated specific surface areas and pore volumes and are, therefore, particularly advantageous for adsorption/release applications. In particular, as far as silica–bMCD aerogels are concerned, it should be pointed out that, to the best of the authors’ knowledge, only two works are currently present in the literature. The first one, by Matias et al. [36], is not a one-pot process since the CDs were grafted on a previously obtained silica aerogel. In the second one, Jiang et al. [37] prepared a hybrid material through a one-pot route, but they did not directly include a pure CD in the synthesis since a more complex molecule (polyrotaxane) was employed.

Finally, both the dense and the aerogel silica–bMCD hybrids obtained in the present study are characterized in terms of their physico-chemical properties (infra-red spectroscopy, thermogravimetry) and structure (X-ray diffraction, electron microscopy) in order to investigate their composition and the interaction between the organic component (bMCD) and the inorganic one (silica).

## 2. Results

### 2.1. Characterization of the Dense Silica–bMCD Sample

**Figure 1:**
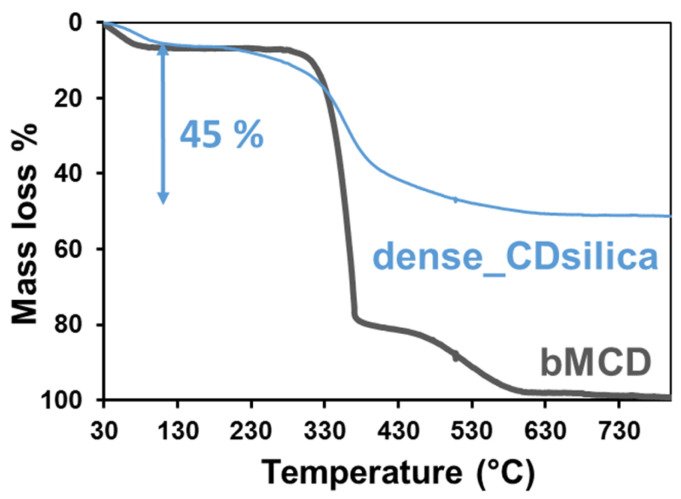
TGA curves of the dense_CDsilica hybrid and of the pure bMCD.

Figure 1 reports the TGA analysis of the dense silica–bMCD hybrid (dense_CDsilica) and, as a comparison, that of pure methyl-β-cyclodextrin (bMCD). Both materials present a slight mass loss below 150 °C, which is followed by steep mass loss between 300 °C and 400 °C and a minor loss between 400 °C and 600 °C, while no relevant phenomena are observed between 600 °C and 800 °C. The first mass loss (below 150 °C) can be ascribed to the evaporation of the physisorbed water; for both samples, this accounts for around 5 wt.%. The second mass loss (between 300 °C and 400 °C) is attributed to the thermal degradation of the organic compound (i.e., bMCD), and the third one (above 400 °C) to its oxidation [38]. If the contribution of water is excluded, the total mass loss due to the presence of organic species (i.e., bMCD) can be calculated between 150 °C and 600 °C. In this range of temperature, the pure bMCD loses 95 wt.% of its original weight, while the dense_CDsilica loses 45 wt.%; in both cases, the mass loss can be ascribed to the presence of bMCD. Indeed, it has to be noted that the main mass loss observed in the dense_CDsilica sample takes place in a range of temperatures (300–600 °C) that is compatible with that of the degradation of pure bMCD. This result suggests that the dense_CDsilica sample contains 45 wt.% of bMCD (corresponding to 0.35 mmol of bMCD per gram of hybrid material), with the remaining mass being silica.

**Figure 2:**
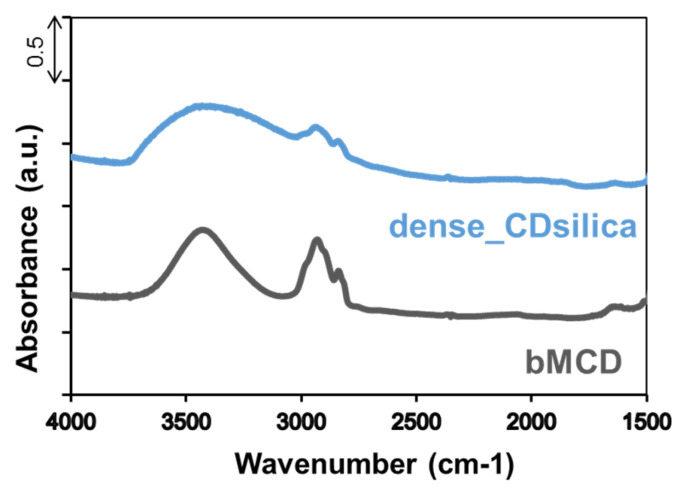
FTIR spectra of the dense_CDsilica hybrid and of the pure bMCD.

Figure 2 shows the FTIR spectra of the dense_CDsilica and pure bMCD. In the spectrum of the pure bMCD, a broad peak is observed at around 3400 cm^−1^ and can be attributed to the stretching vibration of –OH groups [39]. In addition, peaks below 3000 cm^−1^ are present in the bMCD spectrum, due to the stretching of –CH_2_- and –CH_3_ groups [39]. In the dense_CDsilica sample, a broad band is visible between 3600 cm^−1^ and 3200 cm^−1^, which can be ascribed to –OH groups (from both bMCD and silanols of silica) interacting through hydrogen bonds. Moreover, peaks comparable to those of the pure bMCD are present below 3000 cm^−1^. These can again be attributed to the stretching of –CH_2_- and –CH_3_ groups and are further evidence of the presence of an organic component (namely, bMCD) in the hybrid material.

**Figure 3:**
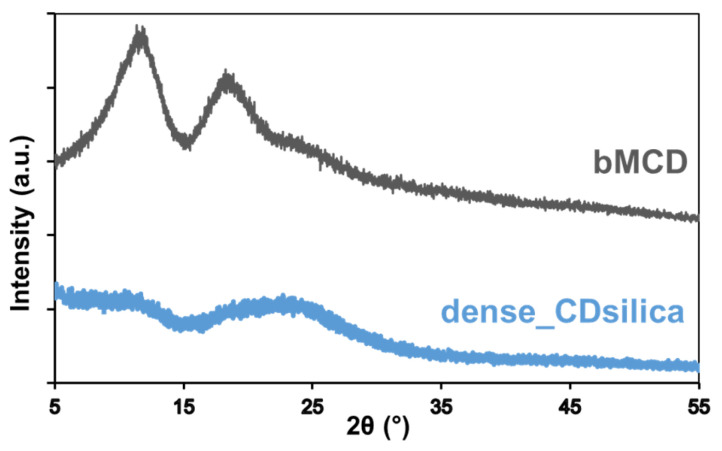
XRD patterns of the dense_CDsilica hybrid and of the pure bMCD.

The XRD patterns of the dense_CDsilica and pure bMCD are shown in Figure 3. Both materials do not present any peak, so revealing a crystalline phase, but rather display the broad halo typical of amorphous phases. The pattern of the pure bMCD is comparable to those reported in the literature [40].

**Figure 4:**
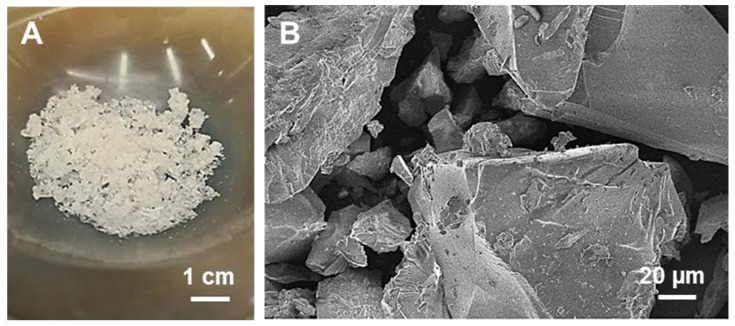
Photo (**A**) and FESEM (**B**) images of the dense_CDsilica hybrid (after manual grinding).

The macroscopic feature and the morphology of the dense_CDsilica hybrid (after manual grinding) can be observed in Figure 4A and Figure 4B, respectively. The hybrid appears as a white and glassy material (Figure 4A). The particles have an irregular and sharp shape with dimensions in the range of some tens of microns; cleavage planes due to the manual grinding are easily visible (Figure 4B).

### 2.2. Characterization of the Aerogel Silica–bMCD Samples

**Figure 5:**
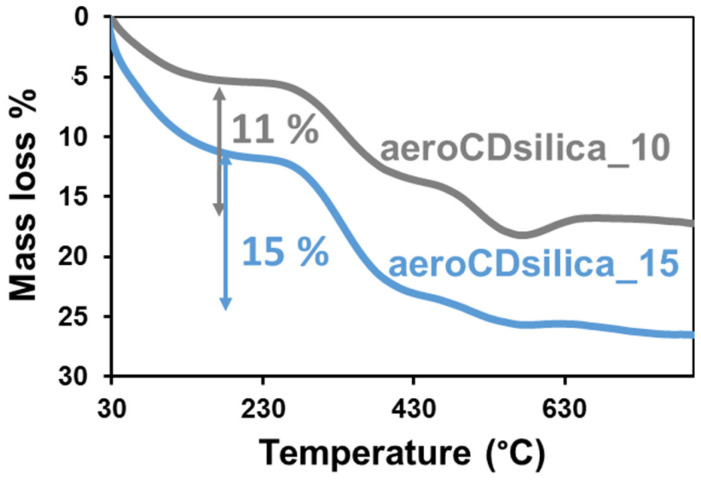
TGA curves of the aeroCDsilica_10 and aeroCDsilica_15 hybrids.

The TGA curves of the two aerogel samples are reported in Figure 5. They differ for the nominal content of bMCD: the sample named aeroCDsilica_15 has a 15 wt.% nominal content of bMCD, while aeroCDsilica_10 has a 10 wt.% nominal content of the oligosaccharide. Both aeroCDsilica_10 and aeroCDsilica_15 present an initial mass loss below 150 °C due to the evaporation of the physisorbed water (5 wt.% and 10 wt.%, respectively). A higher mass loss, attributed to the degradation of bMCD, takes place between 300 °C and 400 °C, which is followed by a final mass loss, ascribed to the oxidation of bMCD [38], between 400 °C and 600 °C. The total mass loss between 150 °C and 600 °C, which is a direct indicator of the bMCD content, accounts for 11 wt.% and 15 wt.% for aeroCDsilica_10 and aeroCDsilica_15, respectively (corresponding to 0.08 and 0.12 mmol of bMCD per gram of hybrid material), suggesting that the real bMCD content is very close to the theoretical one (i.e., no bMCD is lost during the synthesis process). The slight difference between the theoretical and the measured content observed for the aero_CDsilica_10 sample can be ascribed both to instrumental errors (which can account for up to 1% [41]) and to the condensation of silanols (which can cause reduced mass losses at temperatures higher than 500 °C, thus slightly affecting the result [42]).

**Figure 6:**
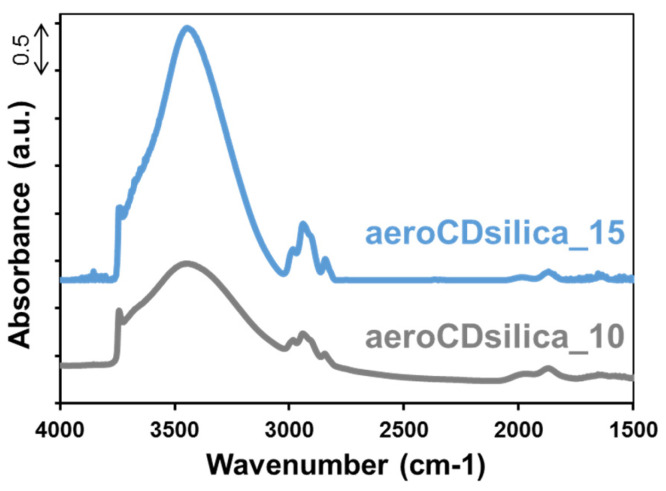
FTIR spectra of the aeroCDsilica_10 and aeroCDsilica_15 hybrids.

Figure 6 reports the FTIR spectra of the aerogels. The peak visible at around 3700 cm^−1^ is attributed to isolated silanols [43,44], while the broad band between 3600 cm^−1^ and 3200 cm^−1^ is due to –OH (from both silica silanols and bMCD) interacting through hydrogen bonds [45]. It is worth noting that the relative intensity of the band due to interacting –OH, both due to silica and bMCD, is higher in the aeroCDsilica_15 sample with respect to that of the aeroCDsilica_10 one. This is coherent with the higher capacity of aeroCDsilica_15 to adsorb water at room temperature, as revealed by the higher mass loss due to the evaporation of the physisorbed water measured by TGA (Figure 5). This difference in intensity can be attributed to a higher bMCD content (i.e., a higher content of –OH due to bMCD) and also to a higher amount of –OH of silanols interacting through H-bonds, since it cannot be excluded that the silanol abundance is different in the two aerogels. Finally, the peaks below 3000 cm^−1^ are attributed to the stretching of –CH_2_- and –CH_3_ groups and, being peculiar in each organic compound, they confirm the presence of bMCD in the hybrid samples [39]. The relative intensity of these peaks is higher for the aeroCDsilica_15 sample, which is coherent with its higher nominal content of bMCD.

**Figure 7:**
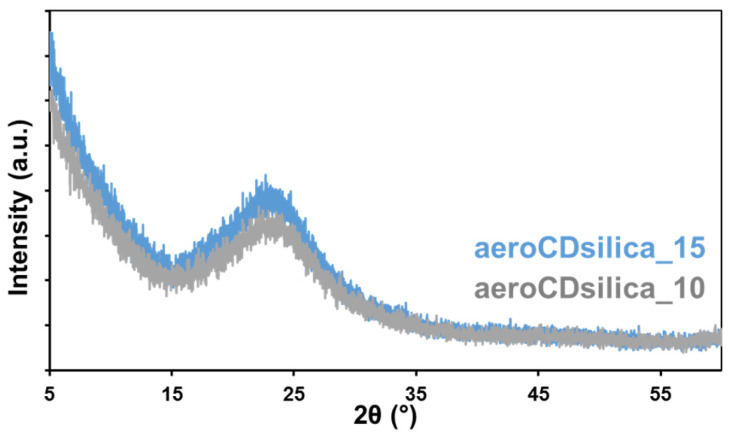
XRD patterns of the aeroCDsilica_10 and aeroCDsilica_15 hybrids.

The XRD patterns of both aeroCDsilica_15 and aeroCDsilica_10 (Figure 7) are characterized by a halo typical of amorphous materials, without any evidence of crystalline phases.

**Figure 8:**
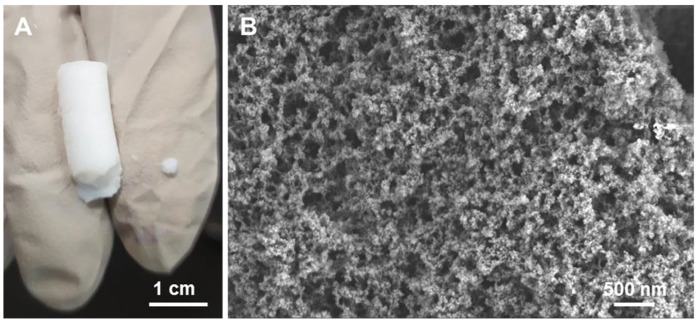
Photo (**A**) and FESEM (**B**) images of aeroCDsilica_10 in monolithic and powder form, respectively.

An example of the macroscopic aspect of one of the monolith aerogels (aeroCDsilica_10) and an SEM micrograph of the powder of the same hybrid (obtained by manual grinding) are reported in Figure 8. Monoliths are whitish cylinders of 10 mm in diameter and around 20 mm in length (Figure 8A). According to these measurements and the weight of the samples, the apparent density of the monolith aerogels (obtained by dividing the mass by the volume) was roughly estimated to be equal to 0.12 cm^3^/g and 0.16 cm^3^/g for the aeroCDsilica_10 and aeroCDsilica_15, respectively. The SEM images of the powder sample reveal a regular morphology with small particles of a few tens of nanometers that are connected to form a porous structure with small inter-particle porosities (of a few nanometers) and bigger pores of tens to hundreds of nanometers (Figure 8B).

**Figure 9:**
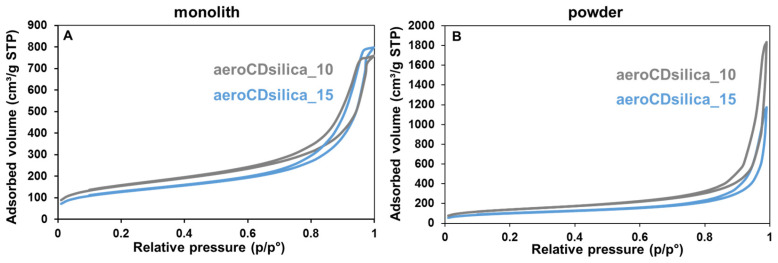
Nitrogen adsorption isotherms of the aeroCDsilica_10 and aeroCDsilica_15 hybrids in the monolithic (**A**) and powder forms (**B**).

Nitrogen adsorption isotherms are shown in Figure 9A,B for the aerogel samples in monolith and powder form, respectively, with the powder obtained by manually grinding the monolith. The monolith samples have a type IV isotherm with a hysteresis H1-H2b loop [46], suggesting that these materials are mesoporous and have a complex pore structure. The powder samples instead present a type II isotherm, typical of inter-particle macroporosity, with a hysteresis loop due to inter-particle condensation [46]. The values of the specific surface area (SSA), pore volume (V_por_) and average pore diameter (Φ pores) obtained from the nitrogen adsorption isotherms are displayed in Table 1. All the SSA values are in the range of 350–550 m^2^/g; the higher values belong to the monoliths and the lower ones to the powders. The pore volumes are around 1.2 cm^3^/g for both the monolithic samples, while they are equal to 2.8 cm^3^/g and 1.8 cm^3^/g for the powders of aeroCDsilica_10 and aeroCDsilica_15, respectively. Finally, the average pore diameter is around 15 nm for both the monolithic samples and around 30 nm for the powders. As could be expected, the addition of inclusions in the silica network causes a decrease in the surface area, as can be noted when the SSA values of aeroCDsilica_10 and aeroCDsilica_15 are compared, with this last sample containing a higher bMCD amount and displaying a smaller SSA. As far as the influence of the grinding procedure is concerned, the samples in powder form possess a smaller surface area but a higher pore volume when they are compared to the corresponding monoliths. The decrease in SSA may be explained by a mechanical collapse of the porous network induced by grinding. The increase in the porous volume, instead, is likely due to the appearance of inter-particle volumes, which can again be ascribed to the grinding process.

To check the stability of the aerogels over time, the samples of aeroCDsilica_10 and aeroCDsilica_15 in powder form underwent a second nitrogen adsorption measurement after 4-month storage in plastic vials in a desiccator at room temperature. The isotherms are comparable to those obtained for the “freshly-prepared” samples (type II isotherm with inter-particle condensation, Figure 9B), and the corresponding SSA and V_por_ are reported in Table 1 with the labels aeroCDsilica_10_4m and aeroCDsilica_15_4m, respectively. For both samples, the pore volume slightly increased over time, while the specific surface area and the mean pore diameter remained constant, which suggests that the sample structure is stable over time and it does not undergo any degradation or collapse.

**Figure 10:**
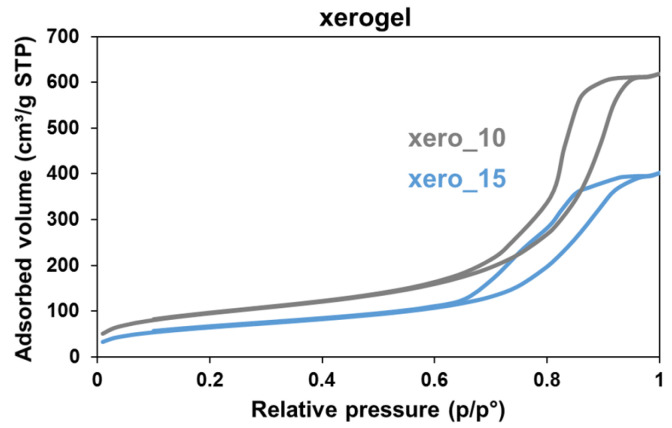
Nitrogen adsorption isotherms of the xero_10 and xero_15 hybrids.

Finally, part of the gel obtained during the sol–gel procedure was not subjected to supercritical drying, but rather, was allowed to dry at room temperature under atmospheric pressure, thus leading to the formation of a so-called xerogel [47]. These samples, which derived from the synthesis process of aeroCDsilica_10 and aeroCDsilica_15, were called xero_10 and xero_15, respectively. The textural properties of xero_10 and xero_15 were assessed through nitrogen adsorption; the results are reported in Figure 10 and in Table 1. Also, in this case, in comparison to the monolith aerogels (Figure 9A), the isotherms of xero_10 and xero_15 are type IV with an H2b hysteresis loop, indicating that these materials are mesoporous with a disordered porous structure. However, when they are compared to the aerogel monoliths and powders, the xerogels possess a significantly lower specific surface area and pore volume. In particular, when compared to the corresponding aerogel powder, both xero_10 and xero_15 have an SSA reduced by around 30% and a V_por_ reduced by almost 70%. In addition, the average pore diameter is reduced by 30 to 50% and the maximum pore diameter is below 20 nm. This result underlines, as expected, the strong influence of the drying procedure, highlighting the role of supercritical drying in preserving the bigger pores of the wet gel, which with traditional drying would collapse, as observed in the xerogel samples [48].

## 3. Discussion and Conclusions

The FTIR analyses of the dense sample (Figure 2) confirm that a silica–bMCD hybrid was successfully obtained. bMCD is included in the material in a high amount (~45 wt.%, Figure 1). The bMCD content (≈3% molar with respect to the Si content) was chosen in order to fall in the range identified by Sawicki and co-workers (2–4% molar) as the best one for obtaining the optimal adsorption of pesticides by CD–silica nanocomposites [31]. The obtained material is amorphous (Figure 3), suggesting good dispersion of the bMCD molecules in the hybrid. bMCD is known to be less prone to crystallization with respect to b-CD [49]; however, it has been reported that some processes (such as kneading and co-evaporation [40]) are able to induce bMCD crystallization. On the contrary, the present synthesis procedure does not induce any crystallization of bMCD, which may impair the complexing ability of the bMCD [49].

The high content of bMCD has excellent promise from the perspective of using such a hybrid for the adsorption or the release of molecules; in fact, the higher the bMCD content, the higher the number of molecules that can be complexed. However, this material is obtained in a bulk, dense form, which confers good mechanical properties to the hybrid (in spite of the high bMCD content), but has the main drawback of lacking high specific surface area and porosity.

To overcome this flaw, a new synthesis procedure was set up in order to obtain silica–bMCD hybrids in monolith aerogel form. This novel one-pot process succeeded in producing, for the first time, silica–bMCD aerogels. These materials possess significant content of bMCD (10–15 wt.%, Figure 5 and Figure 6) in amorphous form (Figure 7) and, above all, are characterized by elevated specific surface areas (450–550 m^2^/g), high pore volumes (1.2 cm^3^/g) and low density (0.12–0.16 cm^3^/g). The aerogel monoliths are mesoporous and possess pores in a wide range of diameters from a few nanometers to some tens of nanometers.

In the aerogel hybrid, according to the TGA analysis, the amount of bMCD accounts for 11–15 wt.% (Figure 5), which is considerably less compared to the content of the dense hybrid (45 wt.%, Figure 1). The diverse bMCD content is coherent with the different structures of the two types of hybrids. Indeed, in the dense_CDsilica sample, the bMCD can be included in the silica network even in a high amount (as long as the percolation threshold is not reached [50]) without compromising the mechanical properties of the hybrid, since the silica structure is dense. On the other hand, the structure of the aerogels is made, by definition, of nano-sized silica particles assembled in thin interconnected chains [51]. This peculiar structure confers excellent insulating properties to the aerogels but is detrimental to the mechanical properties of this class of materials. With these premises, the addition of inclusions such as bMCD particles can further deteriorate the mechanical properties of the aerogel network. This is the reason why when the silica–bMCD aerogels were synthesized in this work, the oligosaccharide content was deliberately lower with respect to that of the dense silica–bMCD hybrids.

Although they are fragile, the hybrid aerogels are stable over time. When ground into powder and stored in a desiccator, their specific surface area and pore volume are constant for months (at least 4, according to the experimental results reported in Table 1), which suggests that the porous structure does not collapse. It is worth mentioning that the grinding process influences the textural properties of the aerogel hybrids, not only by decreasing the SSA and increasing the V_por_, but also by broadening the pore size distribution, thus inducing the formation of larger pores, which may be advantageous for some specific applications (for example, to adsorb macromolecules). As far as the porosity and surface area are concerned, the key role of supercritical drying was confirmed. This step, indeed, is pivotal in maintaining a broad size distribution of pores, thus resulting in SSA and Vpor values that are significantly higher than those of the corresponding xerogels obtained by drying the gels at ambient pressure and room temperature (Figure 10 and Table 1).

Overall, the present work reports different strategies to obtain silica–bMCD hybrid materials with various textural properties, ranging from a dense to a highly porous material (aerogel) and passing through an intermediate system (xerogel). Depending on the different properties, each hybrid can be potentially employed in different applications.

First of all, it is interesting to underline how all the hybrid materials prepared in this work potentially have double functionality: indeed, the bMCD may complex lipophilic molecules, while the silica may preferentially adsorb hydrophilic compounds, which makes these hybrids highly promising for the treatment of complex industrial wastes or for the simultaneous release of different lipophilic and hydrophilic active principles. For example, these kinds of materials could be exploited for the adsorption of phenolic compounds, which contain a hydrophilic hydroxyl group and a hydrophobic aromatic ring very close one to the other [36], or of diphenyl-based pesticides, which possess high affinity for the cavity of CDs [31]. It is worth underlining that the double functionality of these hybrids is obtained by a simple co-precursor method, without requiring the use of silylating steps (which would involve long-term processes and the consumption of organic solvents [52]) to impart a hydrophobic character to the hybrid material.

The dense silica–bMCD hybrid is characterized by a high content of bMCD and a packed structure; these features could be advantageous in applications where, for example, a slow but sustained release is needed. In such a dense system, in fact, an elevated number of molecules could be complexed by the bMCD; however, those molecules would need long diffusion times to be released by the hybrid. A possible application, therefore, could be the release of active principles for the long-term treatment of chronic pathologies.

On the other hand, the aerogel hybrids have a lower bMCD content, but they can be easily and rapidly exposed to the surrounding environment thanks to the high specific surface area and porosity of these materials. Possible applications for these aerogels could be in remediation, in sectors where the fast adsorption of contaminants is required, as well as in rapid drug delivery, when the fast release of active principles is desired to treat acute symptoms.

## 4. Materials and Methods

**Figure 11:**
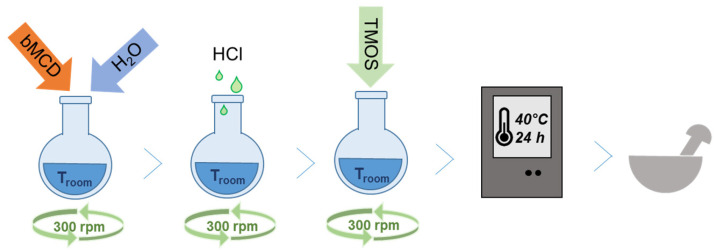
Schematic representation of the synthesis procedure to obtain the dense silica–bMCD sample.

The synthesis of the dense silica–bMCD hybrid (named dense_CDsilica) was performed by adapting the procedure reported by Polarz and Han [53,54]. In particular, 2.0 g of methyl-β-cyclodextrin (bMCD, C_54_H_94_O_35_, Merck Life Science, Darmstadt, Germany) was dissolved in 3.0 g of distilled water (water for chromatography, Merck) under stirring (300 rpm) at room temperature for 10 min. Then, 3 drops of HCl (37%, Sigma-Aldrich, Burlington, MA, USA) were added in order to reach a pH value between 1 and 2. Afterwards, 3.9 mL of tetramethyl orthosilicate (TMOS, SiC_4_H_12_O_4_, >98.0%, Fluka, Charlotte, NC, USA) was added dropwise under constant stirring. The stirring was stopped, and the solution was kept on a heating plate for 2 h at 40 °C, and then, aged for 22 h in an oven at 40 °C. The obtained material was a glassy bulk, which was manually ground (Figure 11).

**Figure 12:**
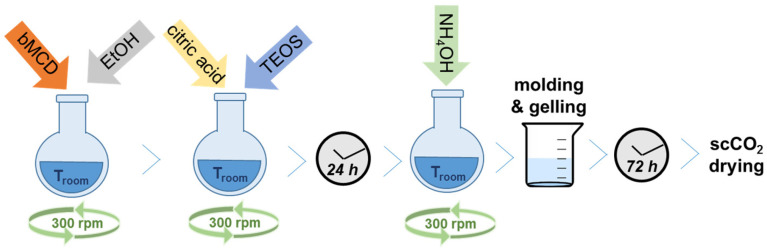
Schematic representation of the synthesis procedure to obtain the silica–bMCD aerogel samples.

As far as the hybrid aerogel preparation is concerned, the procedure reported by Rao et al. [55] was adapted in order to introduce the bMCD in the synthesis batch. Samples with two different contents of bMCD were prepared: one with a 15 wt.% nominal content, named aeroCDsilica_15 (where the number refers to the nominal content of bMCD), and one with a 10 wt.% nominal content, named aeroCDsilica_10. First, a solution of 0.33–0.49 g of bMCD and 9.7 g of ethanol (C_2_H_5_OH anhydrous, Fluka, Charlotte, NC, USA) was stirred (300 rpm) at room temperature for 5 min. Then, 1.9 g of 0.001 M citric acid (C_6_H_8_O_7_, ≥99.5%, Thermo Scientific Chemicals, Pittsburgh, PA, USA) and 6.6 mL of tetraethyl orthosilicate (TEOS, SiC_8_H_20_O_4_, ≥99.995%, Sigma-Aldrich) were added and the solution was left under stirring at room temperature for 24 h. After that time, 1.2 g of 0.5 M NH_4_OH (Sigma-Aldrich) was added, and the solution was stirred for 5 min. Then, the solution was poured into cylindrical glass molds and let to jellify (≈1 h) (Figure 12). The gel was then covered with a saturated solution of bMCD in ethanol and aged for 72 h. The obtained gels were removed from the molds and placed in a 40 mL stainless steel vessel (with an internal diameter of 14 mm). They were first flushed with supercritical CO_2_ (scCO_2_) for 5 min at 40 °C and 120 bar at a flow rate of 10 mL/min to remove the ethanol surrounding the samples. After this, they were dried for 90 min at 40 °C, at 120 bar and 1 mL/min. Finally, the vessel was depressurized at 5 bar/min, and then, allowed to naturally cool down before recovering the samples.

Thermogravimetric (TGA) analyses were carried out using a Setaram TGA (Caluire, France) by heating the samples between 20 °C and 800 °C with a heating rate of 10 °C/min in air flow.

X-Ray diffraction (XRD) data were collected using a Panalytical X’Pert PRO (Cu Kα radiation, Malvern Panalytical, Almelo, The Netherlands), at 40 kV and 40 mA, with a solid-state detector (PIXcel1D). Measurements were performed at high angles (2θ = 5°–60°).

Field emission scanning electron microscopy (FESEM) images were recorded on Pt-metallized specimens using a FESEM Zeiss Merlin (Oxford Instruments, Abingdon-on-Thames, UK).

FTIR spectra were recorded at a resolution of 2 cm^−1^ on pelletized powders (with the addition of KBr only for the dense bMCD–silica sample) using an Equinox 55 spectrometer (Bruker, Billerica, MA, USA) after outgassing the sample at room temperature (residual pressure of 0.1 Pa).

Nitrogen adsorption–desorption isotherms were acquired using an ASAP 2020 Plus analyzer (Micromeritics, Norcross, GA, USA). Samples were degassed at 70 °C in a nitrogen atmosphere at a pressure of 10 mHg for 3 h. The surface area was calculated using the Brunauer–Emmett–Teller (BET) method [56], and pore volume using the Barrett–Joyner–Halenda (BJH) method [57], using desorption isothermal data.

## Figures and Tables

**Table 1:** Textural properties of the hybrid aerogels in the monolithic and powder forms.

Sample	SSA (m^2^/g)	V_por_ (cm^3^/g)	Φ Pores (nm)
aeroCDsilica_10 monolith	550	1.2	15
aeroCDsilica_15 monolith	450	1.2	15
aeroCDsilica_10 powder	490	2.8	30
aeroCDsilica_15 powder	360	1.8	30
aeroCDsilica_10 powder_4m	494	3.2	30
aeroCDsilica_15 powder_4m	375	2.1	30
xero_10	340	0.9	11
xero_15	240	0.6	7

## Data Availability

Data will be shared upon request to the authors.
